# Psychosocial risk factors for dysfunctional beliefs towards motherhood

**DOI:** 10.1192/j.eurpsy.2021.482

**Published:** 2021-08-13

**Authors:** C. Cabaços, D. Pereira, J. Azevedo, M.J. Soares, A. Araujo, A. Macedo, A.T. Pereira

**Affiliations:** 1 Psychiatry Department, Centro Hospitalar e Universitário de Coimbra, Coimbra, Portugal; 2 Institute Of Psychological Medicine, Faculty Of Medicine, University of Coimbra, coimbra, Portugal

**Keywords:** Perinatal Mental Health, Postpartum depression, Dysfunctional beliefs towards motherhood

## Abstract

**Introduction:**

Motherhood-related beliefs are characterized by themes of failure and maternal role idealization. Recent studies found that postpartum depression/PPD is both predicted and a predictor by/for dysfunctional beliefs/DB. Additionally, it is possible that when contextual factors (eg. lack of social support) are present, women may anticipate the parenting experience as being of isolation, which in turn can lead to more dysfunctional attitudes.

**Objectives:**

To explore psychosocial risk factors for motherhood-DB.

**Methods:**

233 women were evaluated in the second trimester (17.05±4.82 weeks) of pregnancy and in the third month (12.08±4.25 weeks) postpartum sociodemographically and psychosocially (years of education, previous children and social support) and the Portuguese validated self-report questionnaires to assess: perinatal depression; perinatal anxiety; perfectionism; negative affect; self-compassion; and repetitive negative thinking (all in T0). The Attitudes Towards Motherhood Scale was administered in the postpartum. When Pearson/Spearmen correlation coefficients proved significant (p<.05), linear/logistic (hierarchic) regression analysis were performed.

**Results:**

Motherhood-DB correlated significantly with all the variables, except for years of education, Other-oriented-Perfectionism and Common-Humanity. Motherhood-DB were significantly higher in women without previous children (p<.05). The final regression model was statistically significant (p<.001) explaining 15% of the Motherhood-DB variance, with Socially-Prescribed-Perfectionism and social support being the only statistically significant predictors. Hierarchic regression showed that even after controlling for social support, SSP significantly incremented the variance in 9%.

**Conclusions:**

Our results highlight the need for preventive approaches to help women understand the origins of their dysfunctional beliefs (perfectionism, the myths of perfect motherhood) and for the promotion of positive cognitions.

**Disclosure:**

No significant relationships.

